# Are the Kessler Psychological Scales suitable for screening for mental disorders in low-threshold mental health services in German-speaking countries?

**DOI:** 10.1192/j.eurpsy.2022.894

**Published:** 2022-09-01

**Authors:** F. Schultze-Lutter, C. Michel, B. Schimmelmann, E. Meisenzahl, N. Osman

**Affiliations:** 1 Heinrich-Heine University Düsseldorf, Department Of Psychiatry And Psychotherapy, Düsseldorf, Germany; 2 University of Bern, University Hospital Of Child And Adolescent Psychiatry And Psychotherapy, Bern, Switzerland; 3 LVR-Klinikum Düsseldorf, Heinrich-Heine University Düsseldorf, Department Of Psychiatrie, Düsseldorf, Germany

**Keywords:** screening, general population, concurrent validity, Mental Disorders

## Abstract

**Introduction:**

The Kessler Psychological Distress Scales (K10 and K6) are used as screening tools to assess psychological distress and are the first-line assessment of need for help in the Headspace services.

**Objectives:**

Thus, we studied the psychometric properties of their German versions in a Swiss community sample to evaluate their potential usefulness to screen for mental disorders or relevant mental problems in low threshold transdiagnostic German-speaking services.

**Methods:**

The sample consisted of 829 citizens of the Swiss canton Bern of age 19-43 years. K10/K6 were validated against Mini-International Neuropsychiatric Interview (M.I.N.I.) diagnoses, questionnaires about health status and quality of life. Receiver Operating Characteristic (ROC) curve analyses were used to test for general discriminative ability and to select optimal cut-offs of the K10 and K6 for non-psychotic full-blown and subthreshold mental disorders.

**Results:**

Cronbach’s alphas were 0.81 (K10) and 0.70 (K6). ROC analyses indicated much lower optimal thresholds than earlier suggested; 10 for K10 and 6 for K6. At these thresholds, against M.I.N.I. diagnoses, Cohen’s Kappa (<=0.173) and correspondence rates (<=58.14%) were insufficient throughout. Values were higher at the earlier suggested threshold, yet, at the cost of sensitivity that was below 0.5 in all but three, and below 0.3 in all but six cases.

**Conclusions:**

For the lack of sufficient validity and sensitivity, respectively, our findings suggest that both K10 and K6 would only be of limited use in a low-threshold transdiagnostic mental health service – comparable to Headspace – for young adults in Switzerland and likely other German-speaking countries.

**Disclosure:**

No significant relationships.

